# American Medical Biography

**Published:** 1866-06

**Authors:** 


### American Medical Biography.

"Dr. J. M. Toner, of Washington, D. C., is engaged in compiling and writing a *Biographical Dictionary* of all deceased American physicians of whom he can collect data of a sufficiently accurate character to enable him to give a brief sketch of their lives and labors.

Physicians and others who have deceased relatives or friends who studied or practised medicine in any part of the United States, and will take the pains to furnish the Doctor with definite facts, comprising full name, birth-place, date of birth and death, education, medical studies, place of graduating, location, success in any particular branch of practice, and whether holding any and what important public positions; if an author, the title of his publications, where and by whom published—will, besides conferring a favor on Dr. Toner, serve the cause of medical literature, of a very attractive and useful kind. It is expected that the collection will reach about ten thousand names."—*Medical and Surgical Reporter.*

We most cordially approve of this proposition, while we regard writing Biographical sketches of living Physicians as an abuse to be corrected.

